# Proton Radiotherapy for Pediatric Sarcoma

**DOI:** 10.3390/cancers6010112

**Published:** 2014-01-14

**Authors:** Matthew M. Ladra, Torunn I. Yock

**Affiliations:** Department of Radiation Oncology, Massachusetts General Hospital, Boston, MA 02114, USA; E-Mail: mladra@partners.org

**Keywords:** sarcoma, pediatrics, protons, ewings, osteosarcoma, rhabdomyosarcoma, chordoma, chondrosarcoma

## Abstract

Pediatric sarcomas represent a distinct group of pathologies, with approximately 900 new cases per year in the United States alone. Radiotherapy plays an integral role in the local control of these tumors, which often arise adjacent to critical structures and growing organs. The physical properties of proton beam radiotherapy provide a distinct advantage over standard photon radiation by eliminating excess dose deposited beyond the target volume, thereby reducing both the dose of radiation delivered to non-target structures as well as the total radiation dose delivered to a patient. Dosimetric studies comparing proton plans to IMRT and 3D conformal radiation have demonstrated the superiority of protons in numerous pediatric malignancies and data on long-term clinical outcomes and toxicity is emerging. In this article, we review the existing clinical and dosimetric data regarding the use of proton beam radiation in malignant bone and soft tissue sarcomas.

## 1. Introduction

Pediatric sarcomas represent a diverse and challenging group of malignancies. Treatment requires a varied, multidisciplinary approach based on histology and location. Adjuvant radiation remains a mainstay of local therapy but is associated with significant acute and long-term treatment morbidity within multiple organ systems and an increased risk of second malignancy [1]. Advances in surgical technique, systemic therapy, and radiotherapy imaging and delivery have increased survival across all sarcoma subtypes. With improved clinical outcomes, greater emphasis becomes focused on reducing long-term toxicity in these young patients. Proton therapy has become a useful tool in the drive to limit acute and late effects.

Proton therapy as large field fractionated treatment was first used in clinical practice in the United States at the Harvard Cyclotron Laboratory in Cambridge, MA beginning in 1974. To date more than 74,000 patients have been treated with proton radiotherapy worldwide. Internationally there are 36 active proton facilities, including 11 within the United States, and additional centers are under development. Currently, the capital cost of constructing a multi-gantry proton center exceeds 100 million dollars. Newer treatment designs in production aim to decrease this cost, and smaller single-gantry facilities are being developed and offered in the range of 20–30 million dollars. At present, the cost ratio of delivering a fraction of proton radiation is 2.4 times that of photon radiation [2], although with decreasing technical costs this ratio is expected to drop significantly.

**Figure 1:**
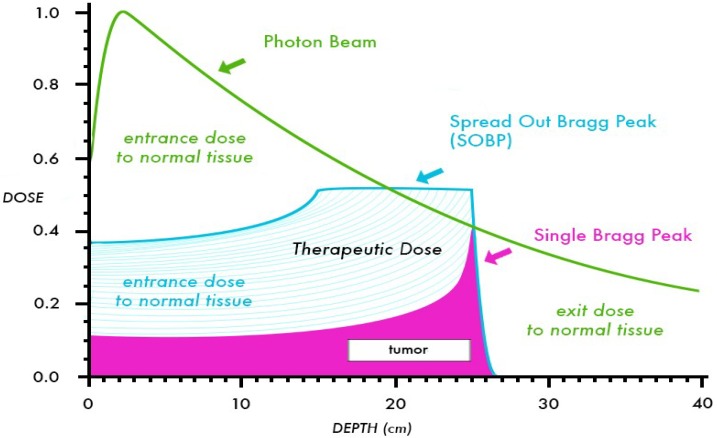
Comparison of depth dose curves for a 10 MV photon beam and a 10 MeV proton beam (shown with and without a SOBP). This figure shows the decreased entrance dose and absence of exit dose for the proton beam in comparison to the photon beam.

Because the relative biologic effectiveness (RBE) of protons is nearly equivalent to that of high-energy X-rays (RBE = 1.1), the interest in protons is based primarily on physical properties that provide favorable dose distributions [3]. Compared to photons, protons deposit a nearly constant quantity of radiation until the end of the beam range, where the majority of energy is released over a short distance called the Bragg Peak, roughly 5–10 mm in length. To cover a target of larger size, beam modulation is used to create a “spread out” Bragg Peak (SOBP) that expands the area of high-energy deposition to cover the desired target length. As opposed to photon beams which have a gradual dose fall off after reaching the target depth in a patient, the Bragg Peak in proton beams leads to a complete absence of “exit dose”, or dose beyond the target volume. A comparison of depth dose curves for a 10 MV photon beam, a 10 MeV proton beam, and SOBP are shown in Figure 1. Through the elimination of exit dose, protons can provide significant advantages over photons by decreasing the dose to adjacent critical structures such as the spinal cord, brainstem, or kidneys, and allowing for increased total dose to be given to the target volume. Further, the “integral dose” or total energy deposited in a patient from radiation therapy is decreased significantly. In dosimetric comparisons, the integral dose per patient treated with protons is decreased by a factor of 2–3 compared to photon therapy [4]. Typically, proton dose is prescribed in cobalt Gray equivalent (CGE) units, using an RBE of 1.1. For example, a proton dose of 50.4 CGE represents an energy deposit equivalent to 45.8 Gy of photon radiation but has an *in vivo* effect of 50.4 Gy, attributable to the increased RBE. For clarity and ease of presentation, the proton doses discussed in this review will be written using “Gy”, but it should be noted that the units represent the CGE.

The potential for reduced secondary malignancy attributable to improved dose distribution with proton beam radiation was examined in a publication by Miralbell *et al*. [5]. Three treatment plans (3D conformal photon, IMRT, and proton) were generated and compared for one patient with medulloblastoma, and four plans (3D, IMRT, proton, and intensity modulated proton therapy (IMPT)), were created for a rhabdomyosarcoma (RMS) of the paranasal sinus. The absolute risks of secondary cancer for each treatment plan was estimated based on dose-volume distributions for the non-target organs using models from the International Commission on Radiologic Protection. Proton beam reduced the expected incidence of radiation-induced secondary cancers for the RMS patient by a factor of more than 2, and for the medulloblastoma patient by a factor of 8 to 15 when compared with either IMRT or 3D conformal radiation. Taken together, these properties provide a strong impetus for the use of proton therapy in children.

## 2. Rhabdomyosarcoma and Soft Tissue Sarcoma

Rhabdomyosarcoma (RMS) represents the most common pediatric soft tissue sarcoma, with approximately 350 new cases per year within the United States [6]. There is a bimodal age distribution, with two thirds of the cases occurring before the age of ten, followed by a second peak in adolescence. These tumors arise in a variety of locations, including the head and neck (35%), genitourinary tract (24%), limbs (19%), and elsewhere (22%), and only one-third are readily resectable [7]. RMS patients are stratified into low, intermediate and high-risk groups based on site of origin, size, histology, lymph node involvement, metastatic spread, and extent of surgical resection [8]. Patients with low risk disease who undergo a complete resection do not need radiation. Low risk patients with a gross total resection and positive margins receive 36 Gy, those with positive nodes 41.4 Gy and those with gross residual disease 50.4 Gy. Intermediate risk patients, those with alveolar histology or those with gross disease at the start of chemotherapy, will receive radiation therapy (RT) regardless of surgical margin status if delayed resection is done. Children with orbital primaries are treated to 45 Gy after biopsy alone. All patients typically receive VAC- or VAI-(vincristine, dactinomycin, and cyclophosphamide or ifosfamide) based chemotherapy.

As many of these tumors arise in close proximity to critical structures in developing children, the incorporation of proton RT into treatment has been explored and the recently closed Children’s Oncology Group (COG) and International Rhabdomyosarcoma Study Group (IRSG) protocols allowed for the use of proton RT to treat patients. At Massachusetts General Hospital (MGH), proton RT has been used to treat pediatric RMS patients with protons since the 1990s. The 3-year outcomes for 57 RMS patients enrolled on a prospective joint proton protocol with MD Anderson Cancer Center (MDACC) between 2005 and 2012 were recently presented in abstract form [9]. Median three-year local control (LC), progression free survival (PFS) and overall survival (OS) were 82%, 75%, and 81% respectively, comparable to published results from the IRS IV/V trials [10,11,12]. The protocol included those with localized disease and patients with metastatic embryonal RMS between the ages of 2–10 years. No patients developed acute or late toxicity of grade 4 or 5 and there were three incidences of grade 3 late toxicity, related to chronic otitis, cataract, and dry eye in head and neck tumors. 

Several smaller site-specific studies using protons in RMS have been published. A retrospective review by Childs *et al*. of 17 consecutive children with parameningeal rhabdomyosarcoma (PM-RMS) that were treated with proton radiotherapy between 1996 and 2005, demonstrated comparable outcomes to that seen with standard photon RT [13]. From the photon literature, local control for children with PM-RMS ranges from 80%–90%, and the 5-year failure free survival (FFS) is approximately 67%, but can drop as low as 52% if a patient has an unfavorable parameningeal site and meningeal impingement [1,8,12]. In the proton study, the median age for this study was 3.4 years and mean dose was 50.4 Gy (range, 50.4–56.0 Gy). The 5-year failure-free survival was 59%, and overall survival was 64%. Patients who had intracranial extension at diagnosis (*n =* 10) had a 5-year FFS of 50% and a 5-year OS of 60%. Patients without intracranial extension (*n =* 7) fared better with a 5-year FFS of 71% and a 5-year OS of 71%. Seven of the 17 patients (41%) had a recurrence at a median time of 10.5 months (range, 7–18.5 months) after diagnosis. Among the seven patients who failed, sites of first recurrence were local only (*n =* 2), regional only (*n =* 2), distant only (*n =* 2), and a single patient failed both locally and distant. Ten patients (59%) were without tumor recurrence at study completion and available for late toxicity evaluation. Among these patients, late effects of multimodality treatment included mild facial hypoplasia (*n =* 7), lack of permanent tooth eruption (*n =* 3), decreased height velocity (*n =* 3), endocrinopathies (*n =* 2) and chronic sinus congestion (*n =* 2). These numbers compare favorably to long term studies of traditional photon based treatment where late toxicity for head and neck patients included hearing loss (17%–20%), learning disabilities (10%–49%), facial/orbital hypoplasia (34%–36%) visual problems (17%–30%), decreased height velocity (30%–61%) and poor dentition (23%–29%) [14,15]. 

A separate dosimetric comparison of proton and intensity-modulated photon radiotherapy for 10 pediatric PM-RMS patients was also published by the same group [16]. Each patient was treated with protons, and the proton treatment plan was then compared to an IMRT plan generated for study purposes. In nine cases, the prescribed GTV dose was 50.4 Gy in 28 fractions. In the remaining case, the prescribed GTV dose was 52.2 Gy in 29 fractions. The median CTV prescription dose was 47.7 Gy (range, 36–52.2 Gy). Comparative beam arrangements were given for each proton and photon plan and on average five beams were used in the proton plans and four were used in the photon plans.

Eight patients in this cohort had radiographic evidence of intracranial extension. Proton treatment yielded equivalent target coverage while significantly decreasing dose to the optic structures (globe, lens, optic nerves, optic chiasm, and retina), the hypothalamic-pituitary axis, the brainstem, temporal lobes, cochlea, lacrimal glands, and parotids. Although direct comparison of clinical outcomes could not be done, some differences between the proton and photon plans were quite striking. With regards to lens dose and cataract risk, only one proton plan had a lens dose higher than 5 Gy, whereas in the photon plans 80% of ipsilateral lenses and 60% of contralateral lenses received above 5 Gy. Cochlear dose associated with hearing loss is minimized at doses less than 32 Gy, and no proton plans had a mean contralateral dose above 27 Gy whereas 50% of photon plans had a contralateral cochlear dose over 32 Gy. Similarly, for contralateral parotid glands where long-term salivary dysfunction begins at 26–30 Gy, the mean proton dose never exceeded 13 Gy whereas the mean dose for the IMRT plans was greater than 26 Gy for 70% of cases. And finally, the mean dose to the hypothalamus was 12 Gy for protons *vs*. 22.4 Gy for IMRT. Merchant *et al*. recently published data showing that a cumulative dose of 16.1 Gy to the hypothalamus was the mean radiation dose required to achieve a 50% risk of growth hormone deficiency at 5 years [17]. 

The proton experience for orbital RMS was reported by Yock *et al*. [18]. Seven children were treated to a median dose of 46.6 Gy with a median follow-up of 6.3 years. Six of the seven patients were without evidence of disease and the remaining child was salvaged with exenteration and stereotactic radiosurgery after local recurrence. The child with local failure had a two unfavorable prognostic factors going into RT; progression during chemotherapy and an age less than 1 year. Late effects of treatment were minimal. All six patients retained good vision in the treated eye and two of the six required drops for lubrication but none demonstrated corneal pathology or dry eye syndrome. All patients did develop mild to moderate orbital bony asymmetry or enophthalmous. None of the patients developed neuroendocrine deficits. In the early IRS trials (I–III), diminished height velocity, a marker for hypothalamic-pituitary dysfunction, was 48%–61% for orbital tumors and in more recent IMRT trials including orbital tumors, neuroendocrine dysfunction has ranged from 3%–10% for the small number of patients analyzed [14,15,19,20]. In the publication, the authors also compared the proton plans used for treatment to 3D conformal plans generated for the study. Proton radiation markedly decreased dose to the brain, temporal lobes, hypothalamic-pituitary axis, and both the ipsilateral and contralateral orbital structures with an average percent savings of 82%–94% for CNS structures and 26%–65% for ipsilateral orbital structures.

Finally, Cotter *et al*. reported the outcomes of for seven children treated with protons for bladder/prostate RMS with a median follow-up of 27 months [21]. Patients had a mean age of 30 months (range: 11–70) and radiation dose ranged from to 36 to 50.4 Gy. Five of seven patients (71.4%) were without evidence of disease with intact bladders at study completion. One patient had a local recurrence in the treatment field, while a second had a local and a distant recurrence. Two of the five patients with intact bladders at the end of treatment reported bladder dysfunction, both of which were attributable to prior surgical procedures. No long-term skeletal or gastrointestinal effects were noted, and all patients were too young to assess sexual function. IMRT plans were created for study purposes and compared to the proton plans used for treatment. Proton radiotherapy showed a statistically significant decrease in mean organ dose to the bladder (median proton dose of 25 Gy *vs*. median IMRT dose of 33.2 Gy; *p =* 0.03), testes (0.0 CGE * vs*. 0.6 Gy; *p =* 0.016), femoral heads (1.6 Gy* vs*. 10.6 Gy; *p =* 0.016), pelvic growth plates (21.7 Gy* vs*. 32.4 Gy; *p =* 0.016), and pelvic bones (8.8 Gy *vs*. 13.5 Gy; *p =* 0.016). There were no significant differences seen in dose to the bowel, prostate, penile bulb, and rectum. 

**Figure 2:**
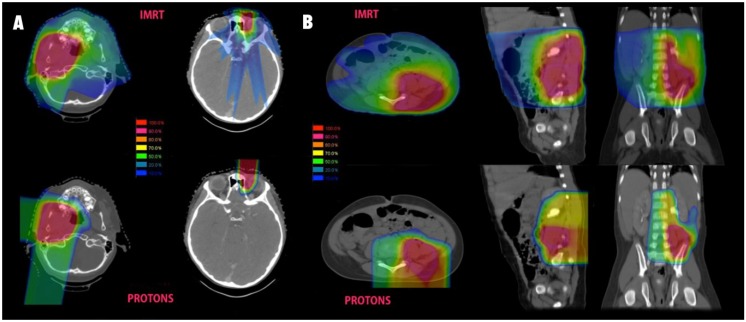
Isodose comparisons for photon and proton plans. (**A**) Typical parameningeal (left) and orbital (right) plans for pediatric RMS. IMRT plans are shown above and proton plans for the same patient seen below; (**B**) A pelvic sarcoma with comparative photon and proton plans shown in three planes.

Pelvic dose sparing in sarcoma treated with protons was also demonstrated in a dosimetric analysis from MD Anderson Cancer Center [22]. A total of three patients with pelvic sarcomas, including one RMS, were planned using various techniques and demonstrated a marked reduction in ovarian dose with protons compared to both 3D conformal and IMRT. Protons allowed for complete sparing of dose to the ovaries while IMRT led to 100% of the ovarian volume receiving 2 Gy and 30% receiving a mean dose of 5 Gy. Significant dose sparing to the pelvic bones and vertebral bodies was also demonstrated with protons, and the mean volume receiving 20 Gy was 13% and 29% with IMRT *vs*. 5% and 9% with protons. IMRT was associated with slightly better bladder sparing in these cases with 2% *vs*. 8% receiving 40 Gy and 0%* vs*. 4% receiving 45 Gy. Some examples of comparative proton and photon plans are shown in Figure 2.

## 3. Non-Rhabdomyosarcoma Soft Tissue Sarcoma

Non-rhabdomyosarcoma soft tissue sarcomas (NRSTS) comprise about 4% of childhood malignancies and affect approximately 500 children under the age of 20 years in the United States each year [6]. The estimated 5-year survival of children and adolescents with non-metastatic NRSTS is roughly 80% [23,24]. The chance of cure is largely affected by surgical resectability, as children with grossly resected non-metastatic tumors have a 5-year estimated survival of 89%, compared with 56% for initially unresected tumors [25]. Radiation as an adjuvant is typically given to unresectable tumors, high-grade tumors less than 5 cm with involved surgical margins, or high-grade tumors greater than 5 cm regardless of margin status, and can be used pre-operatively with chemotherapy for delayed surgery in initially unresected disease. The doses of radiation used in the recently closed COG study for non-rhabdomyosarcomas ARST 0332 ranged from 45 Gy pre-operatively to 55.8 Gy for microscopic residual and 64.8 Gy for gross disease. Anthracycline based chemotherapy can be given for large, high-grade tumors or metastatic disease. A pooled analysis of the European and US experience with pediatric NRSTS from 1980–2005 demonstrated a significant survival advantage to adjuvant radiation after incomplete resection [26]. In the study published by Ferrari *et al*., the 5-year OS was 35% for those that did not receive radiation compared to 69% for those that did receive radiation, and this significance was retained on multivariate analysis.

Few studies of late effects in children and adolescents treated for NRSTS have been published and long-term quality of life and functional outcomes in this population are largely unknown. The recently closed COG protocol ARST 0332 aimed to reduce long-term toxicity by omitting chemotherapy and/or radiation in certain low to moderate risk presentations and will systematically evaluate the acute and long-term sequelae of treatment. Proton therapy was incorporated into the protocol and will be examined. Although to our knowledge there are no existing proton series dedicated to pediatric NRSTS, studies in adult paraspinal NRSTS treated with protons have found comparable local control and excellent toxicity profiles, and total integral dose and mean dose to the organs at risk were consistently shown to be reduced by a factor of 1.3 to 25 in dosimetric studies [27,28].

## 4. Osteosarcoma

Within the United States, approximately 650–700 malignant bone tumors are diagnosed in patients under 20 years, of which 400 (56%) are osteosarcoma (OSA) [29]. Traditionally, osteosarcoma has been viewed as a radioresistant tumor [30] and radiation has been used most successfully in the adjuvant and palliative setting [31]. A role for post-operative radiation in OSA of the head and neck, where gross total resection is frequently unattainable, was demonstrated by Guadagnolo *et al*. in their review of 119 adult and pediatric patients treated at MDACC with photon RT [32]. In the study, 92 (77%) patients underwent surgery alone, and 27 (23%) were treated with surgery and radiation (mean dose = 60 Gy).Compared to surgery alone, RT improved 5-year OS (80% *vs*. 31%), DSS (80% *vs*. 35%) and LC (75% *vs*. 24%) for patients with positive or uncertain resection margins. Despite improvements with adjuvant RT, local control still remains a significant problem and survival after local failure is typically as poor as with metastatic disease. Therefore, there exists great interest in using charged particle therapy to escalate dose to residual tumor and/or resection bed.

The largest experience with protons and osteosarcoma comes from a retrospective series of 55 patients treated at MGH between 1983 to 2009 with proton therapy or mixed photon-proton radiotherapy [33]. This cohort included pediatric and adult patients with a median age of 29 years (range, 2 to 76 years) with OSA of the pelvis, spine, ribs, skull, and facial bones. Most patients had positive surgical margins (49.1%) following surgery and the remainder had biopsy only or minor a resection with residual gross tumor. Radiation dose ranged from 50.4 Gy to 80 Gy; 40% patients received a dose between 60 Gy and 70 Gy and 50.1% received a total dose of ≥70 Gy. Only 11 patients (20%) received proton only RT and the majority of these had primary tumors of the skull and face. Preoperative RT was used in 13% of cases with a dose of 19.8 Gy. MGH often utilizes a low dose pre-operative regimen with the goal of reducing intraoperative tumor seeding while protecting post-operative wound healing. All patients received neoadjuvant anthracycline based chemotherapy.

Clinical outcomes were excellent with 3 and 5 year local control rates of 82% and 72% respectively. Twelve patients failed locally and 10 were within the treatment field while two were considered marginal failures. Of those that recurred locally, 67% were tumors arising from bones of the skull and on multivariate analysis, skull primaries were associated with a hazard ratio of 2.6. At 5 years, DFS was 65% and OS rate was 67%. With regard to late effects, 17 patients (31%) developed grade 3 or 4 toxicity and in 10 patients (24%) the late effects were attributable to radiation. Grade 3 toxicity consisted of severe pain (*n* = 3), diplopia (*n =* 1), immobility of limb (*n =* 2), severe bowel dysfunction due to denervation (*n =* 1), and severe headaches (*n =* 1). Grade 4 toxicity, defined as a loss of organ or organ function, was seen in nine patients. Enucleation after RT was required in four patients, one patient suffered from ipsilateral loss of vision and orbital pain, and one patient suffered from ipsilateral hearing loss and blindness. Two patients were severely handicapped because of immobility or impairment of gait. One patient suffered from extensive structural alteration of the maxillary bone needing repeated adaptation of the prosthesis. Second malignancy was seen in two patients; one patient developed acute lymphocytic leukemia a year and a half after treatment, and the other patient died from a secondary squamous cell carcinoma of the maxilla 16 years after completion of therapy.

In comparing outcomes from this study to prior OSA outcomes published by MGH, the 5-year LC improved from 68% to 72%, despite retaining 40 of the original patients in both studies [34]. The improvement was attributed to the higher RT dose used in the more recent patients and suggests that dose escalation allowed by proton RT will continue to play a role in the advancement of OSA treatment. Further, a meta-analysis done by the authors showed that as doses reached 70 Gy or higher, the benefit of surgical resection prior to RT lost significance. With limited data available, the careful weighing of potential surgical morbidity against the potential late effects of high dose RT should be done before treatment decisions are made.

## 5. Ewing Sarcoma

Ewing sarcoma represents a promising target for improving patient outcomes using proton RT. With 200 cases per year in the US, it is the second most common primary bone tumor after osteosarcoma and comprises 34% of pediatric bone tumors and 3% of all childhood cancers. The majority of patients (approximately 85%) present with primary bone tumors but isolated soft tissue tumors are also observed. Primary sites typically include the pelvic bones (23%), femur (18%), ribs (13%), tibia (10%) and humerus (7%), but tumors can arise from the head, neck, and skull as well. Radiation is used in conjunction with chemotherapy in 60% of Ewing’s cases, either after resection for close/involved margins or as definitive therapy in unresectable disease [35,36,37,38,39]. With 5 year survival for localized disease at an upwards of 60%–70%, reduction of late toxicity to normal tissue has become a major investigational goal [40,41,42,43,44,45]. Further, the need for doses up to 60 Gy and the frequency of primary sites in the pelvis, head and neck, base of skull, and spine make protons an ideal modality in young patients.

The only existing proton series dedicated to pediatric Ewing sarcoma was recently published by Rombi *et al*. The authors retrospectively reviewed 30 pediatric Ewing cases treated with protons between 2003 and 2009 [46]. Most cases arose in the spine (*n =* 14) with other sites including pelvic (*n =* 4), head and neck/orbital (*n =* 4), base of skull (*n =* 3), cranial (*n =* 4) and chest wall (*n =* 1). Gross total resection or near total resection was achieved in only two patients and the majority of patients (17/30) had a biopsy only. Median follow up was 38.4 months all patients received proton RT (median dose 54 Gy, range 45–58 Gy) and chemotherapy. The 3-year rates of LC, EFS, and OS were 86%, 60%, and 89%, respectively. Late sequelae seen consisted of five patients with scoliosis/kyphosis (three mild, one moderate, one severe) and all five had vertebral body primaries with laminectomy prior to RT. One patient with an orbital tumor developed epiphora from canicular stenosis and a left lid lag requiring gold seed placement after surgery and RT, and a second orbital patient developed a corneal ulcer. Endocrine deficiency was seen in two patients treated to cranial/base of skull tumors and high frequency hearing loss was seen in one patient with a head and neck tumor. Four patients also developed secondary hematologic malignancies attributable to chemotherapy.

MGH has also reported outcomes for two patients with Ewing sarcoma involving the sinonasal cavity and anterior skull base [47] and MDACC included one pelvic Ewing patient in adosimetric review of protons [22].

## 6. Chordoma and Chondrosarcoma

Chordomas and chondrosarcomas most commonly arise in adults, with a peak incidence in the fourth and fifth decade [48,49]. Fewer than 5% of these tumors are diagnosed in patients under 20 years of age. In children, chordomas arise more commonly in the spheno-occipital region, rather than the sacral predominance seen in adults, but can be found throughout the spine and sacrum [50]. Chordomas in the pediatric population can behave more aggressively than in adults, presenting with a shorter history of symptoms, a shorter interval to progression after surgery, and a higher rate of metastatic disease, especially in those under 5 years of age [51,52]. Whereas chordomas arise from notochord remnants within the axial skeleton, chondrosarcomas originate from cartilaginous elements within bone and are seen most often in the pelvis and femur but can occur in any number of locations, including the base of skull. The rates of long-term local control following treatment with surgery and radiation are significantly higher with chondrosarcoma than chordoma, ranging from 85%–100%, and systemic therapy can be used in select situations [53,54,55].

Management of chordoma and chondrosarcoma relies heavily upon surgery as primary treatment. Despite advances in microscopic and image-guided neurosurgical procedures, complete resection in these tumors is achieved in only 50%–70% of cases, depending on location. Even with complete surgical resection, local recurrence ranges from 40%–60% [56,57,58]. Following surgery, RT is indicated for the presence of gross or microscopic residual disease, or in cases where violation of the tumor capsule has occurred and there is concern for contamination. Radiation combined with surgery has been shown to reduce local recurrence rates and proton RT has repeatedly demonstrated the ability to safely treat adult skull base patients and achieve higher local control than photon RT [59,60]. In a systematic review of all the major published chordoma series, the benefit of proton RT was demonstrated with an overall 5 year LC/OS of 36%/54% for photons *vs*. 64%/80% for protons [59]. This benefit of RT has also been demonstrated in pediatric photon series and there is some data to suggest children have improved responses to radiation compared to their adult counterparts [51,61]. 

The published data for the use of protons with chordoma and chondrosarcoma is mainly limited to cranio-cervical disease. Halbrand *et al*. published their experience with 30 pediatric patients from l’Institut Curie in Orsay, France [62]. Children with tumors of the base of skull and cervical canal (26 chordoma and 4 chondrosarcoma), were treated with combination photon-proton RT following incomplete surgical resection. The mean total dose was 68.3 Gy although a cone down to the GTV was made after 55 Gy. The maximum dose constraints were maintained at ≤56 Gy to the optic nerves and optic chiasm, ≤64 Gy to the anterior aspect of the brain stem, and at ≤56 Gy to the mid-brainstem. Mean follow up was 26.5 months (range, 5–102 months). Local control was 83% with all five failures seen in chordoma patients. Three of the failures were in field, one was a marginal failure, and one patient experienced seeding along the surgical path. The 5 year OS/PFS was 100%/100% for chondrosarcoma and 81%/77% for chordoma. Toxicity was remarkably low in this series with no child experiencing greater than grade 2 CTCAE acute toxicity. Late toxicity consisted of unilateral hearing loss in one patient and grade 2 endocrine dysfunction in seven patients. Four patients experienced unilateral blindness yet all had reported this lack of vision prior to beginning RT.

Hug *et al*. reported a series of pediatric skull base tumors treated with protons at MGH and Loma Linda that included 10 chordomas and 3 chondrosarcomas, followed for a median of 40 months [63]. Recurrent disease was treated in 6 patients and all patients had evidence of gross residual disease at the time of radiation. All patients underwent resection or biopsy prior to RT. A dose of 78.6 Gy for chordoma and 70 Gy for chondrosarcoma was attempted, although the median dose for the chordoma cohort was 73.7 Gy. More than half of these patients were treated with a mixed photon/proton plan. Normal tissue dose constraints were 60 Gy maximum to optic nerves and optic chiasm, 64 Gy to the brainstem and spinal cord surface, and 53 Gy to brainstem and spinal cord center. Local control was 60% and overall survival was 60% for the chordoma patients. The chondrosarcomas again faired better with all three patients achieving a local control and an overall survival of 100%. No correlation was found between outcome and tumor size, duration of symptoms, or treatment for primary *vs*. recurrent disease, but female patients did have a statistically significant increased rate of progression. Acute effects from treatment were generally related to epilation and mucositis and all resolved after treatment completion. One patient did developed severe headaches within 4 weeks of proton RT and required analgesic treatment with resolution after several weeks. Severe late effects were seen in one chordoma patient after 75.4 Gy who developed cerebellar and brainstem parenchymal damage 16 months after treatment, resulting in right-sided motor weakness and ataxia. The authors note that this patient had three separate neurosurgical resections for progressive disease prior to radiation and that patients with more than two procedures prior to RT have been shown to demonstrate a higher rate of radiation induced brainstem damage [64]. 

The MGH later updated their pediatric series in a clinic-pathologic study published by Hoch *et al.* in 2006 to include 73 base of skull chordoma patients [52]. With a median follow up of 7.25 years, the overall survival rate was reported to be 81% for patients treated with surgery and protons or a combined proton/photon plan. It was noted by the authors that the 5-year survival rate for a similar cohort of 125 adults with base of skull chordomas treated at MGH with surgery and proton beam radiation was 55% [65], potentially illustrating the possibility of improved response to RT in pediatric patients. It should be pointed out that two institutions have subsequently published adult series combining surgery and proton RT for base of skull chordomas with 3 and 5 year survival rates of greater than 80%, though the number of patients in each series were much fewer [66,67]. The authors also performed an in depth histopathologic review of the individual tumors and divided them into “conventional”, “chondroid”, “cellular”, or “poorly differentiated” subtypes and reported the results. Of those who eventually died of disease, 14% were conventional chordoma, 18% were chondroid chordoma, 83% were poorly differentiated chordoma, and none were cellular chordoma. Further the average age of those with poorly differentiated chordoma was significantly younger than the rest of the cohort (4.8 years), and was hypothesized to account for the poor outcomes seen with pediatric chordomas under the age of 5 years. In the pediatric age group, metastatic disease has been documented to occur in up to 57.9% of patients under 5 years of age compared with only 8.5% of children 5 years or older, which the authors again hypothesize could be related to the prevalence of poorly differentiated histology.

Similar results for were reported by Rutz *et al*. at the Paul Scherrer Institute in Switzerland for 10 pediatric patients (six chordomas, four chondrosarcomas) that included extracranial tumors [67]. Tumors treated were found in the brain (*n =* 1), skull base (*n =* 5), cervical spine (*n =* 3), and lumbar spine (*n =* 1). Complete resection was achieved in 3/10 children. Proton dose was 74.0 Gy for chordoma and 63.2–68.0 Gy for chondrosarcoma (median, 66.0 Gy), depending on histopathological grading and concurrent chemotherapy. Median follow-up time was 36 months (range, 8–77 months) and all patients were alive at last follow up without local or distant recurrence. Two patients experienced mild late adverse events with the first patient having neurosensory changes, alopecia and hypoaccusis and the second patient having grade 2 pituitary insufficiency.

## 7. Conclusions

Currently, there is sufficient data to suggest that in most pediatric cancers, sarcoma and otherwise, proton beam radiation delivers plans with superior dosimetric properties, specifically a reduced integral dose and reduced dose to organs at risk. Initial clinical data with protons and soft tissue sarcoma appears to show either equivalence or improvement in outcomes when compared to historical photon controls, and toxicity may be reduced. In the setting of bone and cartilaginous sarcomas a clearer advantage exists, mainly due to the ability to increase total dose while respecting the normal tissue tolerance of adjacent structures. Direct comparisons of proton and photon toxicity are plagued by historical differences in surgical technique, systemic therapy, and radiation delivery, as well as by institutional differences in the collection and scoring of toxicity data. The relatively recent incorporation of proton therapy into the majority of the COG protocols will likely provide the most accurate and applicable assessment of proton benefit. Further, almost all children with sarcoma treated with protons are now being enrolled on institution specific clinical and quality of life protocols, and long-term data will continue to emerge in the coming years. As more centers acquire proton capabilities and comparative costs drop, it is reasonable to expect widespread adoption of protons in a majority of pediatric sarcomas.
